# Management of Patients with IBD and History of Cancer

**DOI:** 10.3390/cancers17071057

**Published:** 2025-03-21

**Authors:** Ilaria Faggiani, Sarah Bencardino, Mariangela Allocca, Federica Furfaro, Alessandra Zilli, Tommaso Lorenzo Parigi, Clelia Cicerone, Virginia Solitano, Laurent Peyrin-Biroulet, Silvio Danese, Ferdinando D’Amico

**Affiliations:** 1 Department of Gastroenterology and Endoscopy I, IRCCS San Raffaele Hospital and Vita-Salute San Raffaele University, 20132 Milan, Italy; faggiani.ilaria@hsr.it (I.F.); bencardino.sarah@hsr.it (S.B.); allocca.mariangela@hsr.it (M.A.); furfaro.federica@hsr.it (F.F.); zilli.alessandra@hsr.it (A.Z.); parigi.tommaso@hsr.it (T.L.P.); cicerone.clelia@hsr.it (C.C.); solitano.virginia1@hsr.it (V.S.); sdanese@hotmail.com (S.D.); 2 INSERM NGERE, Department of Gastroenterology, INFINY Institute, CHRU Nancy, F-54500 Vandœuvre-lès-Nancy, France; peyrinbiroulet@gmail.com

**Keywords:** cancer history, malignancies, inflammatory bowel disease, ulcerative colitis, Crohn’s disease, IBD, UC, CD

## Abstract

The use of advanced therapies, including biologics and small molecules, has become an established clinical practice for the treatment of moderate-to-severe ulcerative colitis (UC) and Crohn’s disease (CD). However, certain patient populations, such as those with a history of cancer, are often excluded from clinical trials evaluating the efficacy and safety of these therapies. This exclusion has historically left clinicians with limited evidence to guide treatment decisions in this high-risk group. Nevertheless, emerging real-world data and updated guidelines increasingly support the safe use of advanced therapies in patients with a prior malignancy. Risk stratification and a multidisciplinary approach, including oncologist input, remain critical in optimizing patient outcomes by assessing both cancer recurrence risk and disease activity. This review aims to provide a comprehensive overview of the current evidence, address existing knowledge gaps, and offer practical insights for the management of IBD in patients with a history of cancer.

## Simple Summary

Crohn’s disease and ulcerative colitis are immune-mediated conditions with increasing prevalence worldwide. In cases of moderate-to-severe disease, treatment often involves immunosuppressive therapies, biologics, and small molecules, which target specific immune pathways to reduce inflammation. For years, there has been considerable debate regarding the safety of these therapies in patients with a history of cancer, given their immunomodulatory effects. However, emerging evidence indicates that these medications can be used safely in this population when managed with careful consideration. A multidisciplinary approach involving both the treating oncologist and gastroenterologist is essential to weigh the potential risks and benefits for each patient. This ensures personalized and safe treatment strategies, providing optimal care while minimizing the risk of cancer recurrence.

## 1. Introduction

The incidence and prevalence of inflammatory bowel disease (IBD), including Crohn’s disease (CD) and ulcerative colitis (UC), are increasing worldwide [1]. These conditions are typically lifelong diseases that often present at a young age and are characterized by a natural history of alternating periods of flares and remission [1]. The primary therapeutic goal is to achieve comprehensive disease control [2]. To achieve this outcome, immunosuppressive and immunomodulatory therapies play a fundamental role.

Over the last two decades, the management of IBD has undergone a paradigm shift with the advent of biologic agents and small-molecule therapies [3]. However, alongside their therapeutic benefits, these treatments have introduced new challenges for clinicians. The use of immunosuppressive and immunomodulatory agents, while highly effective, is associated with potential side effects, some of which remain incompletely understood [4]. One critical area of concern is the management of IBD patients with a prior history of cancer. The lack of clarity has contributed to significant hesitancy in prescribing these treatments to this particular patient population [5]. Concerns arise from the potential for immunosuppressors and immunomodulators (IMMs) to diminish the immune surveillance of tumor cells and chronic latent viral infections, such as Epstein–Barr virus and human papillomavirus, provoking direct DNA alterations in organ cells [6].

In recent years, however, insights from large cohort studies have begun to elucidate the safety profile of biologic agents and small molecules in IBD patients with a previous history of malignancy [7].

This review aims to synthesize the latest evidence on this topic, providing an updated perspective to enhance clinical decision-making and improve the management of these complex cases.

## 2. Materials and Methods

We conducted a comprehensive search of the Pubmed, Embase, and Scopus databases up until 9 March 2025, with the aim of identifying studies regarding IBD and history of cancer. To achieve this, we employed specific search terms such as ‘cancer’, ’history of cancer’, ’malignancies’, and ’history of malignancies’, in conjunction with ’Crohn’s disease’, ’ulcerative colitis’, ’CD’, ’UC’, ’inflammatory bowel disease’, and ’IBD’. We limited our search to articles published in the English language.

Our screening process involved two independent reviewers (IF and SB) who initially assessed titles and abstracts to identify potentially relevant studies. Subsequently, we examined the full texts of these selected articles to determine their eligibility for inclusion. Additionally, we manually scrutinized the reference lists of these articles to ensure that no relevant studies were overlooked during the electronic search. The final inclusion of abstracts and articles was based on their relevance to our research objectives.

## 3. Results

### 3.1. Treating IBD in Patients with a History of Cancer

The term “incident cancer” is used in the medical literature to describe any oncological event following a history of cancer. In the case of a patient with a history of cancer, it can describe local, regional, or metastatic recurrence, as well as the development of a new primary tumor in the same or a different organ [7].

The Cancers Et Surrisque Associé aux Maladies inflammatoires intestinales En France (CESAME) French nationwide observational cohort demonstrated that the overall incidence of cancer was significantly higher in patients with previous cancer than in those without previous cancer with a multivariate adjusted hazard ratio (HR) of 1.9 (95% CI 1.2–3.0; *p* = 0.003) [7]. In particular, among the 16,642 patients with no prior history of cancer at the start of the study, 293 developed a cancer during the observation period, resulting in an incidence rate of 6.1 per 1000 patient-years. In contrast, among the 405 patients with a history of cancer, 23 developed an incident cancer during the observation period, resulting in an incidence rate of 21.1 per 1000 patient-years [7]. On the other hand, a recent meta-analysis by Gupta et al., which included 24,382 patients with immune-mediated inflammatory diseases (IMIDs)—among them, 14 studies focusing on IBD—and encompassing 85,784 person-years of follow-up, supports the reassuring evidence that IMMs snd biologic therapies do not significantly impact the risk of developing new primary or recurrent cancers in patients with a prior history of malignancy [8].

#### 3.1.1. Thiopurines

With the advent of biologic agents and small-molecule therapies, the long-term use of thiopurines in the treatment of IBD has significantly declined. Historically, thiopurines played a fundamental role in the therapeutic algorithm for IBD. However, the latest guidelines for the management of CD and UC recommend the cautious use of thiopurines for maintenance therapy [9,10]. This shift is driven by the increased risk of long-term adverse effects associated with thiopurines, including non-melanoma skin cancer, lymphoma, and genitourinary cancers [11].

Despite the established cancer risks associated with thiopurines, evidence does not suggest an additional risk of incident cancer in IBD patients with a prior history of malignancy, as also specified in the latest ECCO guidelines [11]. A meta-analysis by Shelton et al. [12] provides valuable insights into this issue. Among 3706 IBD patients, accounting for 10,332 person-years of follow-up, there were 539 cases of new or recurrent cancer. The pooled incidence rates (IRs) for patients on no immunosuppressive therapy, thiopurines, or tumor necrosis factor-α (TNF-α) antagonists were 35.7 per 1000 person-years, 37.9 per 1000 person-years, and 48.5 per 1000 person-years, respectively. Importantly, there was no statistically significant difference in cancer risk between these groups (*p* > 0.30 for all comparisons).

The CESAME cohort study further demonstrated that there was no significant difference in the incidence of new or recurrent cancers between patients with a history of cancer exposed to thiopurines or methotrexate and those not exposed to these agents. The rates of incident cancer were 27.0 per 1000 patient-years in patients exposed to immunosuppressants at study entry, compared to 19.2 per 1000 patient-years in those not exposed, with no significant difference between the two groups [7].

The results were corroborated by another large cohort study from the ENEIDA registry. Among 520 IBD patients with a prior history of cancer, 62% were treated with thiopurines alone, 34% were treated with a combination of thiopurines and anti-TNF agents, and 4% were treated with anti-TNF agents alone. The incidence of new cancers was similar between the exposed group (16%) and the non-exposed group (18%), with no statistically significant difference (*p* = 0.53) [13].

#### 3.1.2. TNFα Antagonist

The data available on the use of TNF-α antagonists in patients with a prior history of cancer are primarily derived from retrospective studies. This is because patients with a previous cancer diagnosis are typically excluded from randomized controlled trials (RCTs) conducted for drug approval. As a result, most of the evidence in this area is based on observational and retrospective analyses [11]. One of the first studies to evaluate IBD patients with a history of cancer who were exposed to both antimetabolites (thiopurines, methotrexate) and anti-TNF therapy demonstrated that immunosuppressive treatment following a cancer diagnosis was not associated with an increased risk of either new or recurrent malignancy. The HR was 0.32 (95% CI: 0.09–1.09) for anti-TNF therapy, 0.64 (95% CI: 0.26–1.59) for combination therapy with anti-TNF and an antimetabolite, and 1.08 (95% CI: 0.54–2.15) for antimetabolite therapy alone. However, it is important to note that this was a retrospective study with a limited sample size of only 90 patients [14]. A 2019 meta-analysis by Micic et al. reviewed nine observational studies including 11,679 patients with IMIDs who were exposed to TNF-α antagonists and had a history of cancer. In a subgroup analysis focused on patients with IBD, no increased risk of new or recurrent cancer was observed following exposure to TNF-α antagonists, with an incidence rate ratio (IRR) of 0.68 (95% CI: 0.40–1.16) [15]. Similarly, a retrospective American study involving 184 IBD patients with a prior history of cancer reported an incidence of new or recurrent cancer of 4.2 per 1000 person-years among those exposed to TNF-α antagonists. This study also found no significant association between anti-TNF therapy and an increased risk of cancer recurrence or new cancer development (HR 1.03; 95% CI: 0.65–1.64). Interestingly, the authors noted that, although the study was not able to evaluate risks for specific cancers, 19 patients in the anti-TNF-α group had received this therapy after a melanoma diagnosis, with 4 experiencing a recurrence [16].

Regarding specific cancers, Khan et al. conducted a cohort study focusing on IBD patients with a history of non-melanoma skin cancer (NMSC) within the U.S. Veterans Affairs Healthcare System (VAHS). The study reported the following rates of recurrent basal cell carcinoma (BCC) per 100 person-years: 34.5 for those on active thiopurine therapy, 17.8 for those on anti-TNF therapy alone, and 22.4 for patients receiving combined active thiopurine and anti-TNF therapy [17]. Interestingly, among these groups, only active thiopurine use was significantly associated with an increased risk of recurrent BCC compared to 5-aminosalicylates use alone, with an adjusted hazard ratio of 1.65 (95% CI: 1.24–2.19; *p* = 0.0005) [17].

Also, a recent study from the Rising Educators Academics and Clinicians Helping IBD (REACH-IBD) evaluated 170 patients with a history of recent cancer, with 513 person-years of follow-up. The cohort included patients, 84% of whom had solid-organ malignancies. The incidence rate among patients treated with TNF-α antagonists was 3.4 per 100 person-years, compared to 3.7 per 100 person-years for those treated with non-TNF biologics. There was no significant difference in recurrence-free survival between the two groups (hazard ratio: 0.94; 95% CI: 0.24–3.77) [18].

Based on this evidence, it is clear that TNFα antagonists can generally be used in patients with a history of cancer but that each case should be carefully discussed with oncologists [11].

#### 3.1.3. Vedolizumab and Ustekinumab

Vedolizumab and ustekinumab are considered to be among the safest biologic therapies for patients with IBD. In the meta-analysis by Gupta et al., the rate of incident cancer associated with vedolizumab is reported as 16 per 1000 person-years (95% CI) [8]. This study suggests a numerically lower, albeit statistically insignificant, rate of cancer recurrence with vedolizumab compared to other biologic therapies [8]. Supporting these findings, a retrospective cohort study involving 390 IBD patients with a history of cancer reported that among 37 patients exposed to vedolizumab, 6 developed incident cancer. The adjusted HR was 1.36 (95% CI: 0.27–7.01) [19]. The same study also evaluated 14 patients exposed to ustekinumab, of whom 2 (14%) developed subsequent cancer during a follow-up period of 52 months. Similar to vedolizumab, ustekinumab was associated with an adjusted HR of 0.96 (95% CI: 0.17–5.41), indicating no significant increase in cancer risk [19].

The retrospective study by Vedamurthy et al. analyzed 96 patients who were treated with vedolizumab (VDZ) following a prior cancer diagnosis. The incidence of cancer was 22 per 1000 person-years, with 18 patients either developing new cancers (*n* = 7) or experiencing a recurrence (*n* = 11). Notably, the study concluded that VDZ use was not associated with a significantly increased risk of new or recurrent cancers (HR 1.38; 95% CI: 0.38–1.36) [16].

Also, a French cohort study compared 255 IBD patients with a history of cancer who were treated with TNF-α antagonists to 30 patients treated with vedolizumab. The incident cancer-free survival rates were not significantly different between the two groups (*p* = 0.56). The adjusted cancer incidence rates per 1000 person-years were 41.4 (95% CI: 30.9–57.6) in the anti-TNF group and 33.6 (95% CI: 24.5–48.8) in the vedolizumab group. Notably, in a multivariate Cox model, the choice of the first treatment initiated after cancer diagnosis was independently associated with a reduction in incident cancer-free survival rates [20] (Table 1).

#### 3.1.4. JAK Inhibitors

**Figure 1:**
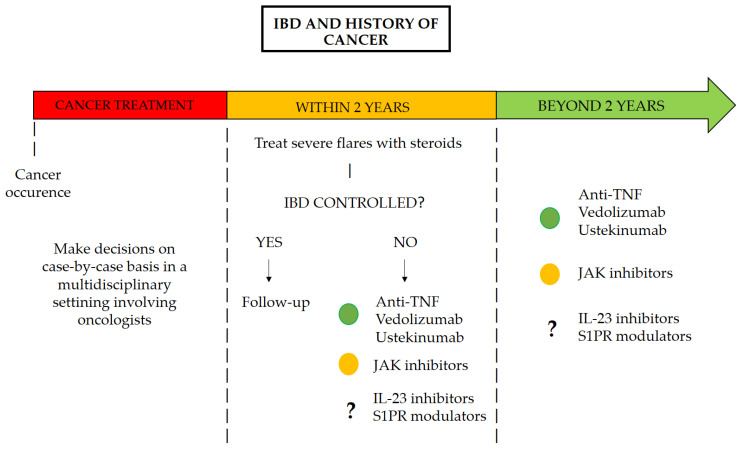
Pathway for the management of patients with IBD with a history of cancer.

The use of Janus kinase (JAK) inhibitors in patients with a history of cancer remains a subject of debate, particularly following the European Medicines Agency (EMA) warning based on findings from the study by Ytterberg et al. on tofacitinib in patients with rheumatoid arthritis [24,25]. In this randomized, open-label, noninferiority trial, Ytterberg and colleagues demonstrated that the incidence of cancer was higher among patients exposed to tofacitinib compared to those receiving TNF-α inhibitors, with an HR of 1.48 (95% CI: 1.04–2.09) [25]. Although this has not been proven in patients with IBD, there is currently insufficient evidence to refute it. In fact, no prospective or retrospective studies have evaluated the safety of JAK inhibitors in this population, and the latest meta-analyses do not include studies evaluating JAK inhibitors [8]. An interim analysis of the ongoing Safety of Immunosuppression in a Prospective Cohort of Inflammatory Bowel Disease Patients With a History of Cancer (SAPPHIRE) registry is the only study to date that has included JAK inhibitors in its evaluation [5]. The final cohort consisted of 305 patients, of whom 210 (69%) were exposed to immunosuppressive therapy during follow-up, while 95 (31%) were not. The incidence rate of new or recurrent cancer among patients who were never exposed to immunosuppression after their cancer was 2.58 per 100 person-years (95% CI: 1.24–4.75). In contrast, patients who received any immunosuppressive therapy had an incidence rate of 4.78 per 100 person-years (95% CI: 3.35–6.62). Notably, among the cohort, 13 patients were exposed to JAK inhibitors, with an incidence rate of new or recurrent cancer of 8.58 per 100 person-years (95% CI: 1.04–31.00) [5]. There is therefore not enough evidence to date to conclude whether or not JAK inhibitors can be used in patients with a history of cancer [11]. Figure 1 presents a potential pathway for the management of patients with IBD who have a history of cancer.

## 4. Discussion

The concern regarding the potential impact of biologic therapies on cancer risk originates from the biological mechanisms underlying their targets. For TNF-α antagonists, the theoretical concern arises from the dual role of TNF-α in malignancy. On the one hand, TNF-α exerts an antitumor effect by inducing apoptosis in malignant cells, raising the concern that inhibiting this cytokine may impair tumor surveillance, potentially causing the recurrence or rapid progression of cancer. On the other hand, TNF-α can facilitate tumor growth by promoting the survival and proliferation of neoplastic cells through the nuclear factor-κB signaling pathway [26,27,28]. This paradox highlights the complexity of TNF-α in the context of malignancy and underscores the need for careful consideration when prescribing TNF-α inhibitors to patients with a history of cancer. In contrast, the mechanism of action of vedolizumab is more selective, as it targets the gut-specific α4β7 integrin. This selective action is associated with a reduced risk of systemic immunosuppression, potentially making it a safer therapeutic option for patients with a history of cancer [29]. Unfortunately, there are still limited data available regarding the safety of recently approved molecules such as selective IL-23 inhibitors in patients with IBD. However, reassuring evidence regarding their safety profile has been derived from their use in other immune-mediated diseases [30]. The established safety profile of ustekinumab, an IL-12 and IL-23 inhibitor, supports the assumption that selective IL-23 inhibitors may also be safe in this patient population [18,23]. Reassuring data on the safety of JAK inhibitors are also emerging from cohorts such as the SAPPHIRE registry [5]. On the other hand, data on S1P receptor modulators remain limited. This drug class, particularly ozanimod and etrasimod, has only recently received EMA approval for the treatment of ulcerative colitis, and the registrational trials did not include patients with a recent history of cancer [31,32].

Regarding the timing of biologic initiation following a cancer diagnosis, current guidelines generally recommend a waiting period of at least two years before considering biologic therapy [11]. However, this recommendation is largely based on expert opinion rather than high-quality evidence, as prospective data on this topic are scarce. The optimal timing of biologic therapy remains an area of uncertainty, and treatment decisions should be individualized, taking into account both the activity of IBD and the patient’s oncologic history.

Multidisciplinary discussions involving gastroenterologists and oncologists are essential in this context. These meetings are crucial to assess the activity or remission status of the neoplasia and to determine whether biologic therapy is warranted based on the activity of IBD and the risk of cancer recurrence. The stratification of patients based on factors such as the type of prior malignancy, time since diagnosis, and current cancer-free status should inform therapeutic decisions [33]. High-risk patients may require more conservative approaches, whereas low-risk individuals might be candidates for biologic therapy earlier in the course of their IBD management.

An additional challenge involves the lack of data on the use of dual therapy in patients with a history of cancer. Dual therapy is often required in cases with extraintestinal manifestations or refractory disease. However, the absence of studies evaluating the safety and efficacy of dual therapy in this context leaves clinicians without clear guidance, highlighting a need for further research [34].

Finally, the available evidence on biologic therapy in patients with IBD and a history of cancer is predominantly derived from retrospective cohort studies. While these studies provide valuable insights, their inherent limitations, including potential biases and lack of standardization, underscore the need for prospective, randomized controlled trials and real-world data. Addressing these gaps in the literature is essential to optimize the management of IBD in patients with a history of cancer. The I-CARE program [4], along with the TREAT [35] and ENCORE [36] registries, are all valuable sources of long-term safety data. These registries will ultimately provide definitive insights into the safety profile of advanced treatments in IBD.

## 5. Conclusions

The management of IBD in patients with a history of cancer remains a complex clinical challenge, with decisions requiring the careful consideration of cancer recurrence risk, IBD activity, and the safety profile of available therapies. Significant gaps remain, particularly regarding the timing of therapy initiation, the role of dual therapy, and the safety of newer agents. Multidisciplinary collaboration and patient stratification are essential in individualized care. Prospective studies and real-world data are needed to guide evidence-based treatment strategies.

## Figures and Tables

**Table 1:** Incidence of new or recurrent cancer in patients with IBD and a history of cancer: results from available cohort studies. PYs, person-years; IMMs, immunomodulators; Anti-TNF, anti-Tumor Necrosis Factor; IBD, inflammatory bowel disease; VDZ, vedolizumab; UST, ustekinumab; NMSC, non-melanoma skin cancer.

Study	Year	Study Design	Type of Prior Cancer	Medication Groups	Patients, *n*	Results
Beaugerie [7]	2014	Prospective observational cohort	Mixed *	Thiopurine/methotrexate	405	Rate of new cancer: 13.2/1000 PYs in patients who were not taking IMMs at the time of study entry vs. 23.1/1000 PYs in patients treated with IMMs; *p* > 0.05 Rate of recurrent cancer: 6.0/1000 PYs in patients who were not taking IMMs at the time of study entry vs. 3.9/1000 PYs in patients treated with IMMs; *p* > 0.05
Onali [21]	2014	Retrospective cohort	Mixed ^£^	Anti-TNF, thiopurines	15	None of the 15 IBD patients treated with IMMs after the diagnosis of cancer showed the recurrence of cancer or had a cancer-related death
Poullenot [22]	2014	Retrospective cohort	Mixed ^$^	Anti-TNF	79	79 cases of IBD patients with previous malignancy: 15 (19%) patients developed incident cancer (8 recurrent and 7 new cancers). Crude incidence rate of new or recurrent cancer: 84.5 per 1000 PYs
Axelrad [14]	2015	Retrospective cohort	Mixed ^%^	Anti-TNF, thiopurines/methotrexate	333	The incident rate of cancer (new or recurrent): 2.46/100 PYs in patient exposed to Anti-TNF 3.63/100 PYs in patient exposed to Anti-TNF+ thiopurines/methotrexate 5.75/100 PYs in patient exposed to thiopurines/methotrexate 5.4/100 PYs in controls
Manosa [15]	2019	Systematic review and meta-analysis of observational studies	Mixed ^&^	Anti-TNF, thiopurines	520	The incident rate of cancer (new or recurrent): 19.6 per 1000 PYs (95% CI: 12.7 28.9) in non-exposed patients 26.2 per 1000 PYs (95% CI: 20.56 33.11) in exposed patients HR 51.68; 95% CI: 51.03–2.73; P: 0.03
Khan [17]	2020	Retrospective cohort	NMSC	Anti-TNF, thiopurines/methotrexate	518	BCC occurrences: 12.8 per 100 PYs in 5-ASA use only 34.5 per 100 PYs in active TP use 19.3 per 100 PYs in past TP use and no anti-TNF use 25.4 per 100 PYs in anti-TNF use after previous TP use 17.8 per 100 PYs in only anti-TNF use 22.4 per 100 PYs in active anti-TNF and TP use
Vedamurthy [16]	2022	Retrospective cohort	Mixed ^^^	Anti-TNF, vedolizumab	463	Rate of incident cancer: 22 per 1000 PYs in patients on VDZ No increase in the risk of new or recurrent cancer with VDZ (HR 1.38; 95% CI: 0.38–1.36) or anti-TNF therapy (HR 1.03; 95% CI: 0.65–1.64) when compared to no IMMs
Hasan [23]	2022	Retrospective case-control	Mixed ^#^	Anti-TNF, vedolizumab, ustekinumab	341	The incident rate of cancer (new or recurrent): 0.4 per 100 PYs with VDZ (HR 0.18; 95% CI: 0.03–1.35) 1.8 per 100 PYs with UST (HR 0.88; 95% CI: 0.25–3.03) 0.7 per 100 PYs with anti-TNF (HR 0.47; 95% CI: 0.20–1.12)
Poullenot [20]	2022	Retrospective cohort	Mixed ^@^	Anti-TNF, vedolizumab, thiopurines/methotrexate	534	The incident rate of cancer (new or recurrent): 47.0 per 1000 PYs for patients receiving no IMMs 36.6 per 1000 PYs in the anti-TNF cohort 33.6 per 1000 PYs in the VDZ cohort [*p* = 0.23]. Incident-cancer free survival rates were not different between patients receiving anti-TNF and those receiving VDZ [*p* = 0.56]
Holmer [18]	2023	Retrospective cohort	Mixed ^¥^	Anti-TNF, vedolizumab, ustekinumab, thiopurines/methotrexate	170	The incident rate of cancer (new or recurrent): 3.4 per 100 PYs among patients treated with TNF-α antagonists 3.7 per 100 PYs for those treated with non-TNF biologics

* Cancer of the colon and rectum, prostate, kidney, small bowel, ear, nose, and throat, thyroid gland, lung, melanoma, testicle, anus, liver, bladder, and other sites, NMSC, non-Hodgkin lymphoma, and Hodgkin disease. ^£^ Not specified. ^$^ Gastrointestinal, dermatologic, and hematologic malignancies and other solid malignancies (breast, prostate, and bladder). ^%^ Gastrointestinal, dermatologic, and hematologic malignancies and other solid malignancies. ^&^ Gastrointestinal, dermatologic, hematologic, and solid-tumor malignancies. ^^^ Gastrointestinal (subtypes: colorectal cancer, small bowel, neuroendocrine, and non-luminal), hematologic, dermatologic, and solid-tumor malignancies. ^#^ Gastrointestinal, dermatologic (melanoma and non-melanoma), hematologic, and solid-tumor malignancies. ^@^ Breast cancer, non-melanoma skin cancer, lymphoma, melanoma skin cancer, leukemia, sarcoma, myeloma, and prostate, kidney, lung, thyroid, uterine, cervical, urothelial tract, head and neck, ovarian, testicular, brain, esophagus, adrenal, liver, and neuroendocrine tumors. ^¥^ Solid-organ (breast, colon, prostate, renal, thyroid, and others) and hematologic malignancies and melanoma.

## Data Availability

No new data were generated or analyzed in support of this research.
